# Development of a novel postoperative adhesion induction model in cynomolgus monkeys with high reliability and reproducibility

**DOI:** 10.1038/s41598-025-88022-3

**Published:** 2025-02-27

**Authors:** Kenji Nakagawa, Masaki Yamazaki, Hiromi Tanimura, Narumi Sakaguchi, Miho Kohara, Izumi Sato, Masahiro Azuma, Ayako Nishimoto-Kakiuchi, Atsuhiko Kato, Takehisa Kitazawa, Ryo Konno, Tadashi Sankai

**Affiliations:** 1 Research Division, Chugai Pharmaceutical Co. Ltd., 216 Totsuka-cho, Totsuka-ku, Yokohama, Kanagawa 244-8602 Japan; 2 Translational Research Division, Chugai Pharmaceutical Co. Ltd., 216 Totsuka-cho, Totsuka-ku, Yokohama, Kanagawa 244-8602 Japan; 3 Tsukuba Primate Research Center, National Institutes of Biomedical Innovation, Health and Nutrition, 1-1 Hachimandai, Tsukuba-shi, Ibaraki 305-0843 Japan; 4 Translational Research Division, Chugai Pharmaceutical Co. Ltd., 2-1-1 Nihonbashi-Muromachi Chuo-ku, Tokyo, 103-8324 Japan; 5 Department of Obstetrics and Gynecology, Jichi Medical University Saitama Medical Center, 1-847 Amanumacho, Omiya-ku, Saitama-shi, Saitama 330-8503 Japan

**Keywords:** Animal disease models, Chemokines

## Abstract

Postoperative adhesions frequently occur following abdominal surgical interventions, leading to serious morbidities and requiring new therapeutic strategies. The development of new therapeutic agents to reduce postoperative adhesions needs animal models that closely mirror human pathophysiology. In this study, we established a novel surgical adhesion model in cynomolgus monkeys, which are characteristically similar to humans. Our model reliably and reproducibly developed adhesions. Histopathological analyses revealed that monkeys undergoing our novel surgery method exhibited changes consistent with those in monkeys that underwent open abdominal surgery. Furthermore, the cellular components of the adhesion tissue in our monkey model reflected those reported in human adhesion tissue. Furthermore, time-course transcriptomic analyses showed that our model accurately recapitulates the well-known progression cascade of postoperative adhesions. In addition, it identified the upregulation of gene that is absent in rodents. We expect our novel surgical method to be a promising tool for elucidating the detailed biology of postoperative adhesions and for assessing new therapeutic treatments with high translatability to human biology.

## Introduction

Postoperative adhesions (PA) are abnormal fibrous bands that form between the peritoneum and visceral organs or between visceral organs themselves following surgical interventions. PA occur in more than half of patients who undergo open abdominal surgery and are associated with serious morbidities such as small bowel obstruction, chronic pain, and female infertility^1,2^. Despite advancements in surgical techniques, PA remain a serious concern, especially with open surgery. It has been reported that the healthcare cost of PA accounts for $1.3 billion annually in the United States^3^.

Various barrier materials have been developed and evaluated to prevent PA formation, with some materials approved for clinical use^4^. However, no specific barrier material has gained widespread clinical adoption due to various drawbacks, including difficulties in preparation and application^5,6^. As a result, there is a pressing need for new strategies to mitigate PA.

PA are believed to result from excessive wound healing processes and follows a cascade of events: surgical trauma induces inflammatory responses, such as the release of proinflammatory cytokines, which in turn attract immune cells such as neutrophils and macrophages to the injury site. Local bleeding induced by surgical trauma and inflammatory responses initiates the coagulation cascade, leading to fibrin deposition. Excessive fibrin deposition serves as the scaffold for fibroblast proliferation, extracellular matrix (ECM) production, and angiogenesis, culminating in PA formation^6,7^.

To prevent PA, the molecular or cellular mechanisms driving these processes are being investigated as therapeutic targets. Rodents including rats and mice are the most widely used animal species for PA research^8^. PA induction in rodent models is commonly achieved by injuring tissues such as the cecum and uterus, combined with a peritoneal insult^8^. Using these models, recent studies have shown that immune cells, including neutrophils, contribute to PA progression, and that targeting these neutrophils prevents PA formation^9,10^.

However, while rodent PA models are well-established and robust, there are genetic and anatomical differences that limit the direct translation of findings from rodents to humans. For example, genomics research has shown that the genes involved in immune responses, such as chemokine activity, differ in rodents and humans^11,12^.

Cynomolgus monkeys (*Macaca fascicularis)* share closer genomic and anatomical similarities with humans, making them a more relevant model for PA research^13^. However, there are currently no standard PA models in cynomolgus monkeys, and there is little information on PA formation in non-human primates.

In this study, we aimed to develop a novel surgical method that can reproducibly induce PA in cynomolgus monkeys. We adopted common surgical procedures—incision, abrasion, and suturing—to induce PA in cynomolgus monkeys and evaluated its reliability and reproducibility. To validate our novel PA induction surgery method, we compared time-dependent histopathological changes in cynomolgus monkeys undergoing PA induction surgery to those observed in monkeys undergoing conventional open abdominal surgery, specifically cesarean sections (C-sections), which are known to occasionally result in adhesions^14^. Additionally, we conducted RNA sequencing analysis to elucidate the detailed transcriptional changes following PA induction surgery and to evaluate whether our novel model recapitulates the reported PA progression cascade.

## Materials and methods

### Animals

The animal studies were conducted using cynomolgus monkeys in the breeding colony of Tsukuba Primate Research Center (TRPC) at the National Institute of Biomedical Innovation, Health and Nutrition (NIBIOHN), Ibaraki, Japan. Cynomolgus monkeys were originally brought from the Philippines, Indonesia, and Malaysia. Although colonies were sometimes crossbred, the monkeys were basically bred within a colony of animals from the same geographic origin. The monkeys which were used for this study were third to fourth generation descendants of the imported monkeys. This study was conducted using six female and nine male cynomolgus monkeys of 6–19 years old in the breeding colony of TPRC from 2017 to 2023. The environment of the animal room was set with 25 ± 3 °C room temperature, 60 ± 5% relative humidity, and a 12 h light-and-dark cycle. The animals were given water ad libitum and fed daily with 70–100 g of commercially available solid food (CMK-2; CLEA Japan, Inc.) and 100 g apples.

### Ethical approval

These studies were approved by the Institutional Animal Care and Use Committee of the NIBIOHN (approval number: DS28-53R6, DS30-37R7). All monkeys used in this study were cared for according to procedures approved by the Animal Care and Use Committee of the NIBIOHN, which reviews study plans according to the guidelines in Japan specified in the ‘Ministry of Health, Labor and Welfare: Basic Policies for the Conduct of Animal Experimentation’. In addition, protocols for all experiments involving animals complied with the guidelines set by the same institute for the care, use, and biological hazard countermeasures of laboratory animals. The study is reported in accordance with ARRIVE guidelines.

### Sample acquisition from cynomolgus monkeys that underwent cesarean sections

Three pregnant female monkeys were used in this study. Under general anesthesia, a midline incision was made, and immediately after the incision, a sample of the abdominal wall was collected as a 0 h sample. At 6 h, 7 days, and 30 days after C-sections procedures, monkeys were anesthetized, and a midline incision was made again and the abdominal wall at the laparotomy site was sampled.

For C-section procedures, general anesthesia was induced via intramuscular administration of a mixture containing ketamine hydrochloride (10 mg/kg; Ketalar, Daiichi Sankyo Propharma Co., Ltd.) and atropine sulfate hydrate (0.05 mL/kg; ATROPINE SULFATE Injection, Nipro ES Pharma Co., Ltd.). Additionally, local anesthetics lidocaine (Xylocaine, Sandoz Pharma K.K.) and bupivacaine (Bosmin, Daiichi Sankyo Co., Ltd.) were subcutaneously injected at the planned incision site. Immediately following fetal delivery, inhalational general anesthesia was administered using sevoflurane (Sevoflurane Inhalation Solution, Viatris Healthcare G.K.). For sample acquisition surgery, general anesthesia was induced via intramuscular administration of a mixture containing ketamine hydrochloride and atropine sulfate.

Following surgery, Cefazolin Sodium (Nichi-Iko Pharmaceutical Co., Ltd.) as an antibiotic and Buprenorphine Hydrochloride (NISSIN, Nissin Pharmaceutical Co., Ltd.) as an analgesic were intramuscularly administered for 3 days after surgery.

Collected samples were placed in 10% Formalin Neutral Buffer Solution (NBF, FUJIFILM Wako Pure Chemical Corporation).

### Surgically induced postoperative adhesions in cynomolgus monkeys

Three female and nine male monkeys were subjected to postoperative induction surgery. PA are reported to form in various organs, including the abdominal wall and uterus^15^. Therefore, we conducted PA induction surgery in abdominal walls and uteruses (Supplementary Fig. S1A).

Monkeys were given general anesthesia by intramuscular administration of a mixture of ketamine hydrochloride (Ketalar, Daiichi Sankyo Propharma Co., Ltd., Tokyo, Japan) and xylazine hydrochloride (Seractal, Bayer Yakuhin, Ltd., Osaka, Japan), after which a midline incision was made. Then, the abdominal walls were gently folded by forceps. Two 10 mm incisions were made on the abdominal wall, approximately 20 mm to the left and right of the midline incision site (Supplementary Fig. S1B Left). These incisions were abraded five times with gauze and sutured with four stitches each with Vicryl Rapide suture 4–0 (Supplementary Fig. S1B Right). In female monkeys, 10 mm incisions were also made on the uterus (Supplementary Fig. S1C Left), and the injured sites were abraded five times with gauze and sutured with four stitches using 4–0 Vicryl Rapide suture, similar to the abdominal wall incisions (Supplementary Fig. S1C Right). Finally, the abdomen was sutured closed. Following surgery, these monkeys underwent the same postoperative treatment as those that had undergone cesarean sections.

### Laparoscopic observation

Monkeys were anesthetized with the same protocol used for PA induction surgery, after which a small midline abdominal incision was made, through which a laparoscope was inserted. Next, a high-flow insufflation unit was used to insufflate carbon dioxide gas into the peritoneal cavity and inflate the abdomen. A small ventral incision was then made, through which a bar marked with a scale was inserted. After the laparoscopic observation, the two incisions were sutured closed with Vicryl Rapide suture 4–0. Following the laparoscopic observation, the monkeys underwent the same postoperative treatment as those that had undergone cesarean sections.

### Acquiring samples from monkeys with surgically induced postoperative adhesions

Monkeys were anesthetized with the same protocol used for PA induction surgery, after which a midline incision was performed. The abdominal wall was sampled immediately after the incision as a control sample (0 h sample). After collecting the 0 h sample, PA induction surgery was performed on the abdominal wall, and the abdomen was sutured closed.

At 6 h (four male monkeys), 7 days (two male monkeys), and 28 days (two male monkeys) after surgery, monkeys were anesthetized with the same protocol used for PA induction surgery. For tissue sampling and the subsequent histopathological analysis, some of the animals were then euthanized via intravenous administrations of pentobarbital. The euthanasia method is an acceptable method of euthanasia for non-human primates per the recommendations of the AVMA Guidelines for the Euthanasia of Animals. Subsequently, an abdominal midline was incised and sampling of the PA-induced areas of the abdominal wall. Adhesed tissues were also sampled at 28 days after surgery. The collected samples were divided into two parts: one part was placed in RNAprotect Tissue Reagent (QIAGEN N.V.), and the other part was placed in 10% NBF. Following the surgery, all monkeys underwent the same postoperative treatment as those that had undergone cesarean sections.

### Histopathological evaluation

Samples fixed in 10% NBF were processed following standard procedures to produce formalin-fixed paraffin-embedded (FFPE) blocks. The histopathological evaluation of monkeys that underwent cesarean sections was conducted using one animal at each time point (6 h, Day 7, Day 30) including 0 h, and that of monkeys with surgically induced PA was conducted using two animals at each time point (6 h, Day 7, Day 28) including 0 h. Thin sections prepared from the FFPE blocks were subjected to hematoxylin and eosin (HE) staining, immunohistochemical (IHC) staining and in situ hybridization (ISH). IHC was performed with antibodies to alpha-smooth muscle actin (α-SMA, 1A4, 760-2833, Ready to use, Roche Diagnostics), CD31 (JC70, 760-4378, Ready to use, Roche Diagnostics), CD68 (KP-1, 790-2931, Ready to use, Roche Diagnostics) and Mesothelin (EPR4509, ab133489, 1 μg/mL, abcam) using a Ventana BenchMark XT automated stainer (Roche Diagnostics) according to the manufacturer’s recommendations. Briefly, the sectioned tissues were deparaffinized, heat pretreated in Cell Conditioning 1 (CC1) for antigen retrieval at 95–100 °C for 0–64 min, and then incubated with the primary antibodies for 16–32 min at 37 °C. The slides were incubated with a secondary antibody followed by the application of HRP Universal Multimer (Roche Diagnostics). Antibodies were detected using DAB and slides were counterstained with hematoxylin II (Roche Diagnostics). ISH was performed for *CXCL8*. The procedure was carried out according to the manufacturer’s recommended procedure with *CXCL8* probe (Mfa-IL-8, 433801, Advanced Cell Diagnostics, ACD) and RNAscope 2.5 HD assay Kit-BROWN (322310, ACD). The histopathological evaluation was conducted by a certified pathologist (MY).

### RNA sequencing and analysis

Total RNA was extracted from the abdominal walls in the monkeys using the RNeasy Fibrous Tissue Mini Kit (QIAGEN N.V.). RNA sequencing was conducted at Takara Bio Inc.

The raw sequencing reads were processed using the following bioinformatics pipeline: STAR (version 2.7.10a)^16^ for mapping the reads to the Macaca fascicularis reference genome (RefSeq GCF_012559485.2), and RSEM (version 1.3.3)^17^ for transcript quantification and generating TPM values using the annotation file (NCBI Macaca fascicularis Annotation Release 102). The quality of the sequencing reads was assessed using FastQC [vesion 0.11.9] (https://www.bioinformatics.babraham.ac.uk/projects/fastqc/) and confirmed to be sufficient.

### Differential expression analysis and enrichment analysis

Differential expression analysis was performed using DESeq2 (version 1.42.1) with adjusted-*p* values < 0.05 and |log2FC| ≥ 1. Due to the limited annotation of the cynomolgus macaque genome, Gene Ontology (GO) enrichment analysis of the differentially expressed genes was conducted using the enrichGO function in the clusterProfiler R package (version 4.10.1) with human annotations. Pathway enrichment analysis was performed using the msigdbr package (version 7.5.1) to query human gene sets in the Molecular Signatures Database (MSigDB)^18^. Plots were generated using ggplot2 [version 3.5.0] in R [version 4.3.2].

## Results

### Time-course analysis of histopathology in monkeys following cesarean sections

**Fig. 1:**
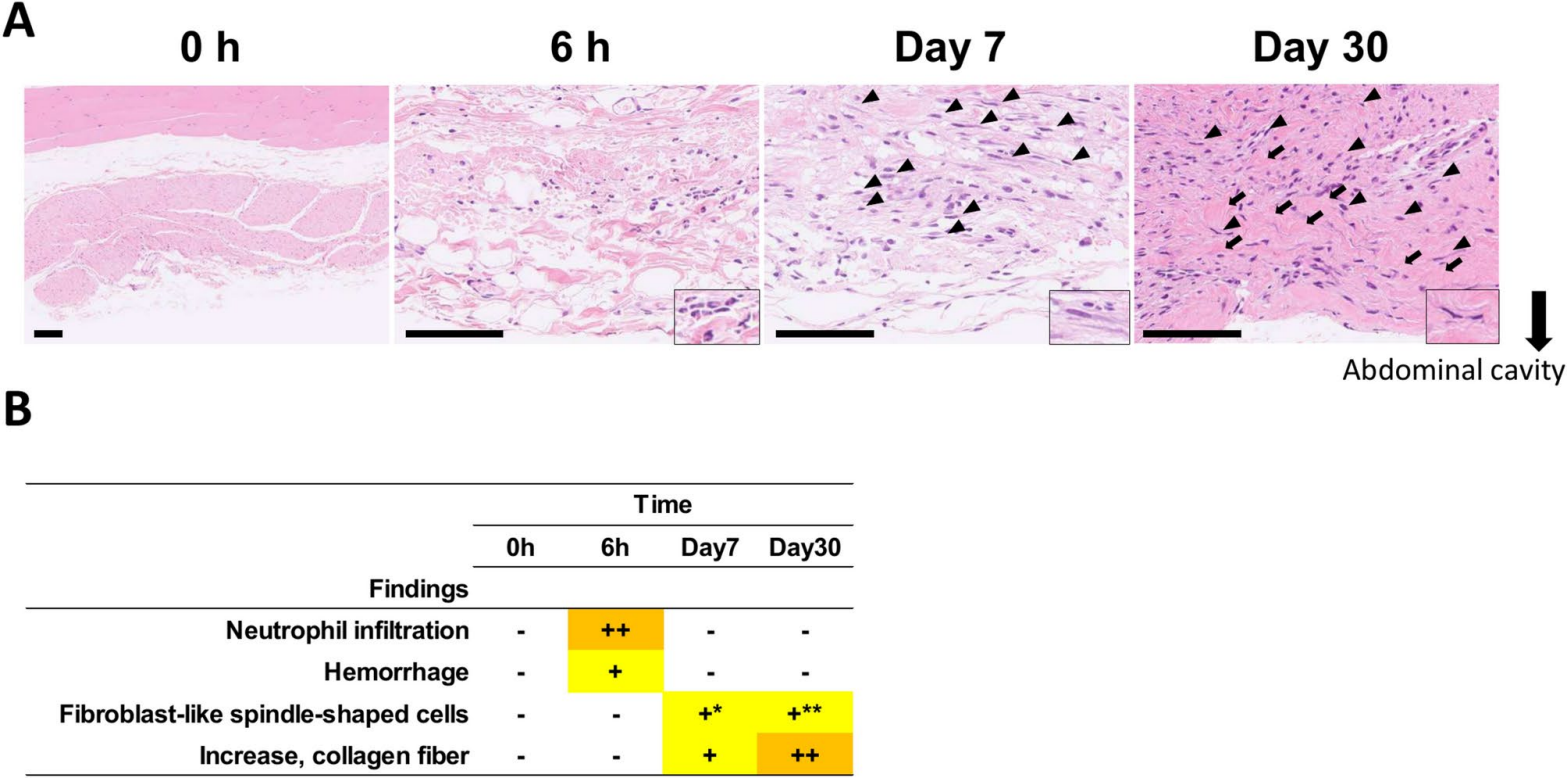
Histopathological analysis of the abdominal walls from monkeys following cesarean sections (C-sections). (**A**) Representative images of hematoxylin and eosin (HE) staining at the site of injured abdominal wall of monkeys following C-sections at the indicated time points post-operation. Insets show neutrophils, fibroblast-like spindle-shaped cells with abundant cytoplasm and thinner fibroblast-like spindle-shaped cells at 6 h, Day 7 and Day 30 respectively. Arrowheads, fibroblast-like spindle-shaped cells; Arrows, collagen fiber. The arrowheads and arrows indicate representative cells or structures. Scale bars, 100 μm. (**B**) Histopathological findings of the abdominal walls from monkeys following C-sections. Grade: -, no abnormality; +, slight; ++, moderate; *, with abundant cytoplasm; **, thinner cytoplasm than Day7.

To evaluate the time-course histopathological changes after open abdominal surgery, we performed histopathological analysis of abdominal walls at midline incision sites in monkeys that had undergone C-sections. Hemorrhage and numerous neutrophil infiltrations were observed 6 h after the C-section surgery (Fig. 1A,B). Additionally, 7 days after the C-section surgery, fibroblast-like spindle-shaped cells with abundant cytoplasm were predominant, and 30 days after the incision, thinner fibroblast-like spindle-shaped cells accompanied by increased collagen fiber were abundant (Fig. 1A,B).

### Evaluation of postoperative adhesion formation in the monkeys following postoperative adhesion induction surgery

**Fig. 2:**
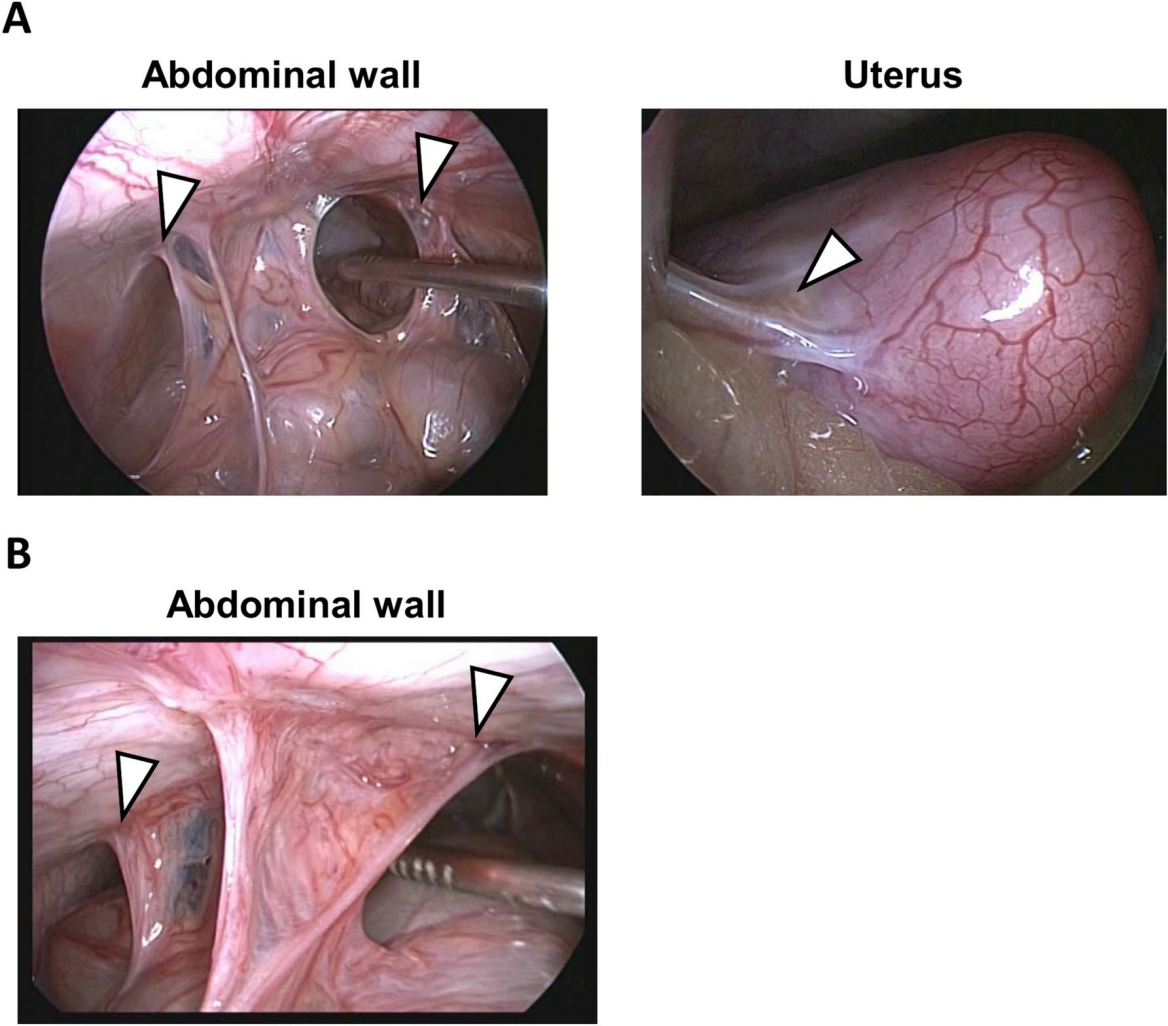
Evaluation of postoperative adhesions (PA) formation in the monkeys following PA induction surgery by laparoscopic observation. (**A, B**) Representative images of laparoscopic observation of PA at abdominal walls (Left) and uterus (Right) in the female monkeys at 26 days after PA induction surgery (**A**) and abdominal walls in the male monkeys at 28 days after PA induction surgery (**B**). Arrowheads indicate the site of injury.

We conducted surgically induced adhesion in the three female cynomolgus monkeys (Supplementary Fig. S1) and performed laparoscopic observations to evaluate PA formation. The laparoscopic evaluation confirmed the presence of PA between the omentum and the injured abdominal walls or uterus at 26 days after PA induction surgery (Fig. 2A). We confirmed that all three female monkeys developed PA.

Subsequently, we also conducted PA induction surgery in three male monkeys. PA induction surgery on abdominal walls was performed, and the presence of PA between the omentum and the injured abdominal walls was confirmed in all three monkeys at 28 days after PA induction surgery (Fig. 2B).

### Time-course analysis of histopathology in the monkeys following PA induction surgery

**Fig. 3:**
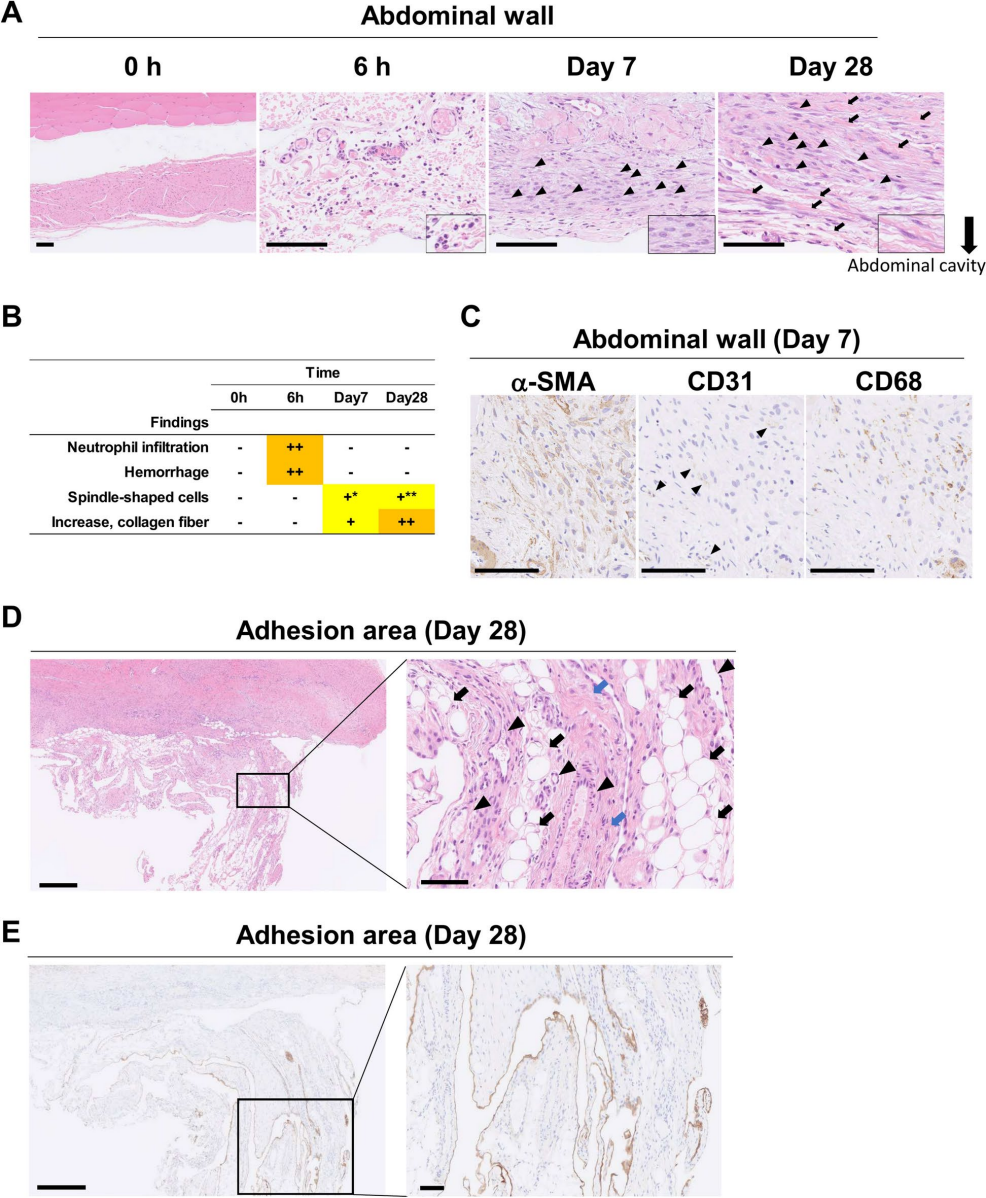
Histopathological analysis of injured abdominal walls and adhesion areas in postoperative adhesion (PA) induced monkeys. (**A**) Representative images of hematoxylin and eosin (HE) staining at the site of injured abdominal wall of monkeys following PA induction surgery. Insets show neutrophils, fibroblast-like spindle-shaped cells with abundant cytoplasm, and thinner fibroblast-like spindle-shaped cells at 6 h, Day 7 and Day 28, respectively. Arrowheads, fibroblast-like spindle-shaped cells; Arrows, collagen fiber. The arrowheads and arrows indicate representative cells or structures. Scale bars, 100 μm. (**B**) Histopathological findings of injured abdominal wall of monkeys following PA induction surgery. Grade: -, no abnormality; +, slight; ++, moderate; *, with abundant cytoplasm; **, thinner cytoplasm than Day 7. (**C**) Representative images of immunohistochemical (IHC) staining on Day 7 for alpha-smooth muscle actin (α-SMA) (Left), CD31 (Center), CD68 (Right) at the site of the injured abdominal wall in monkeys following PA induction surgery. Arrow heads indicate CD31 positive cells. Scale bars, 100 μm. (**D**) Representative images of HE staining at the adhesion area in monkeys following PA induction surgery at 28 days after surgery. Arrows, adipose tissue; Arrowheads, vessel; Blue arrows, spindle-shaped cell with collagen fiber. Scale bars, 500 μm (Left) and 100 μm (Right). (**E**) Representative images of IHC staining for mesothelin. Scale bars, 500 μm (Left) and 100 μm (Right).

We performed time-course histopathological analyses of the injury sites on the abdominal walls in monkeys following PA induction surgery. Numerous neutrophil infiltrations accompanied by hemorrhage were observed at the site of injured abdominal wall 6 h after the surgery, and spindle-shaped cells with abundant cytoplasm were predominant at 7 days after the surgery (Fig. 3A,B). At 28 days after the surgery, the presence of spindle-shaped cells accompanied by increased collagen fiber was observed (Fig. 3A,B). The spindle-shaped cells were thinner than those at day 7. Notably, these histopathological changes were similar to those observed in monkeys that underwent C-sections (Fig. 1A,B).

IHC staining of abdominal walls at day 7 revealed that the majority of the spindle-shaped cells were α-SMA-positive fibroblasts, with some CD31-positive small blood vessels also present. CD68-positive macrophages were also confirmed (Fig. 3C).

Furthermore, histopathological evaluation of the adhesion area itself was performed in the monkeys that had undergone the PA induction surgery at day 28. The cells constituting the adhesion area included spindle-shaped cells with collagen fiber, blood vessels (arteries and capillaries), and adipose tissue (Fig. 3D). We also detected that the mesothelin-positive cells were lined with the adhesion area following PA surgery (Fig. 3E).

### Time-course transcriptome analysis of the monkeys following PA induction surgery

**Fig. 4:**
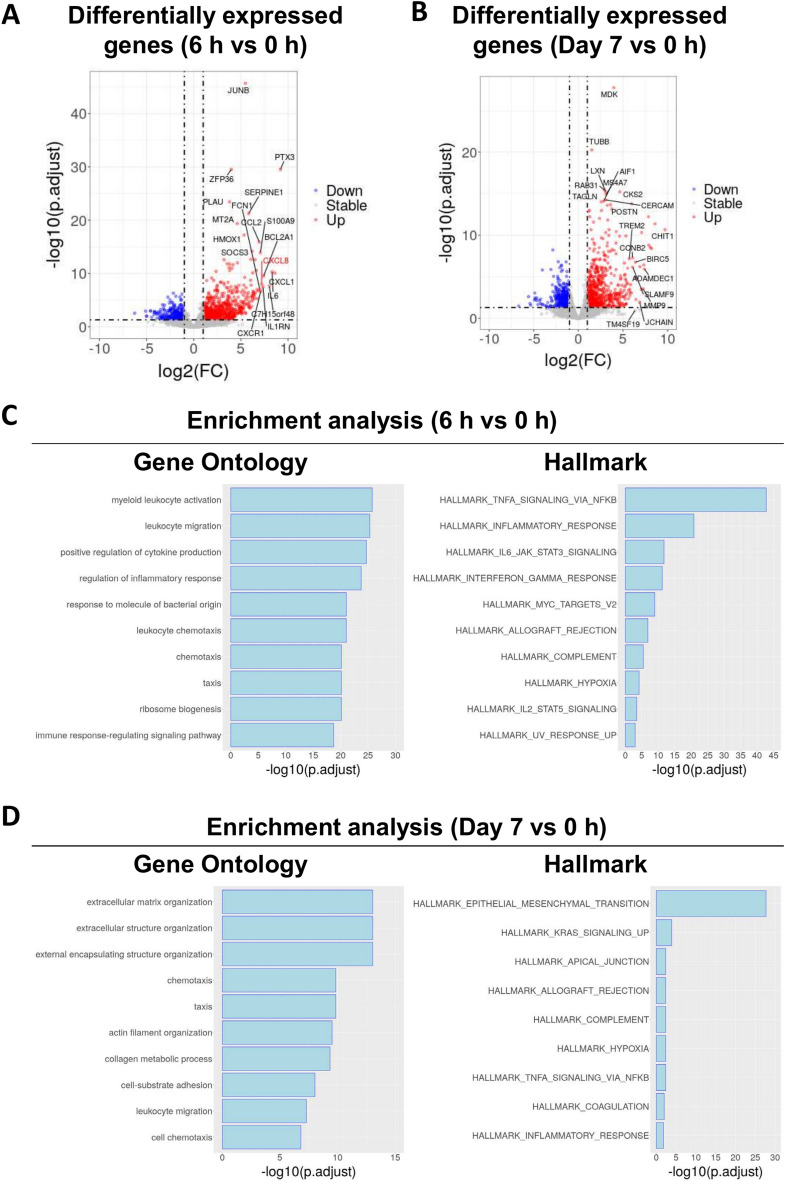
Differential expression analysis and enrichment analysis of monkeys following postoperative adhesions (PA) induction surgery at 6 h and Day 7. (**A, B**) Volcano plot of the gene expression profiles between injured abdominal walls at 6 h (**A**) or 7 days (**B**) after surgery and 0 h samples. Differentially expressed genes (DEGs) with log2 fold change (FC) ≥ 1 and adjusted p-value < 0.05 are in red and DEGs with log2 FC ≤  − 1 and adjusted *p*-value < 0.05 are in blue. Gene symbols show top 10 DEGs based on log2 FC or adjusted p value. (**C, D**) Bar charts showing the top 10 Gene Ontology (GO) terms (Left) and all significant terms for Hallmark (Right) of the upregulated DEGs at 6 h (**C**) or 7 days (**D**) after surgery.

To determine the gene expression profile in the injured abdominal walls before and after PA induction surgery, we performed RNA-seq analysis. First, we performed differential expression analysis between 0 h and each time point. At 6 h post-surgery, 704 genes were significantly upregulated (Fig. 4A). Among them, inflammatory cytokines and chemokines such as *IL-6*, *CXCL1*, *CCL2*, and chemokine receptors like *CXCR1*, as well as the genes involving fibrinolytic system like *SERPINE1* (PAI-1) and *PLAU* (uPA), were drastically upregulated (Fig. 4A). Notably, *CXCL8* (IL-8), a chemokine not present in rodents but crucial in human inflammatory responses^19^, showed significant upregulation at 6 h post-surgery (Fig. 4A). The expression of *CXCL8* was also validated by ISH on the injury sites of the abdominal walls at 6 h (Supplementary Fig. S2). And at day 7, 734 genes were significantly upregulated (Fig. 4B).

To identify the key gene expression programs associated with PA induction, we conducted enrichment analyses using Gene Ontology (GO) and Hallmark gene sets from the Molecular Signatures Database (MSigDB) on the upregulated differentially expressed genes (DEGs). At 6 h, GO analyses showed enrichment in many gene sets related to inflammation, including myeloid leukocyte activation, leukocyte migration, and cytokine production (Fig. 4C, Left). Hallmark gene sets analysis also showed the enrichment of gene sets related to inflammation (TNF-α signaling, inflammatory response, IL6 signaling, IFN-γ signal and hypoxia) at 6 h (Fig. 4C, Right).

Similar analyses conducted for DEGs at day 7 revealed different enriched pathways compared to the 6 h samples. GO analysis at day 7 identified extracellular matrix organization and extracellular structure organization as the most enriched gene sets (Fig. 4D, Left), while Hallmark analysis indicated that epithelial mesenchymal transition was the most significantly enriched gene set (Fig. 4D, Right).

## Discussion

PA remain a significant clinical challenge, causing serious complications for patients. Effective therapeutic drugs for PA treatment are urgently needed. Although rodents such as rats and mice are commonly used for PA research, genetic and anatomical differences between rodents and humans limit the direct translation of findings. To address this gap, we successfully established a novel PA model in cynomolgus monkeys with high reliability and reproducibility. Our novel PA model showed time-dependent histopathological changes consistent with those in monkeys that underwent conventional open abdominal surgery (C-sections) highlighting its validity. Furthermore, time-course RNA-seq analysis revealed that inflammatory responses were activated in the early stage, whereas genes associated with extracellular matrix remodeling were upregulated at a later stage, indicating that our model well recapitulated the PA progression cascade.

In this study, we adopted common surgical procedures—incision, abrasion, and suturing—to induce PA in cynomolgus monkeys. To ensure consistency, we standardized the incision lengths, number of abrasions, and number of sutures, thereby establishing a stable and reproducible method for PA induction. To evaluate the formation of PA, we employed laparoscopic observation, a minimally invasive technique that allowed for the accurate evaluation intra-peritoneal adhesions. Laparoscopic observations revealed that all monkeys, both male and female, developed PA, demonstrating the reproducibility of our surgical method. We also confirmed that PA were formed between injured sites (both abdominal walls and uteruses) and the omentum, which is consistent with clinical findings where the omentum is the most frequent attached site in patients suffering from PA^15^.

**Fig. 5:**
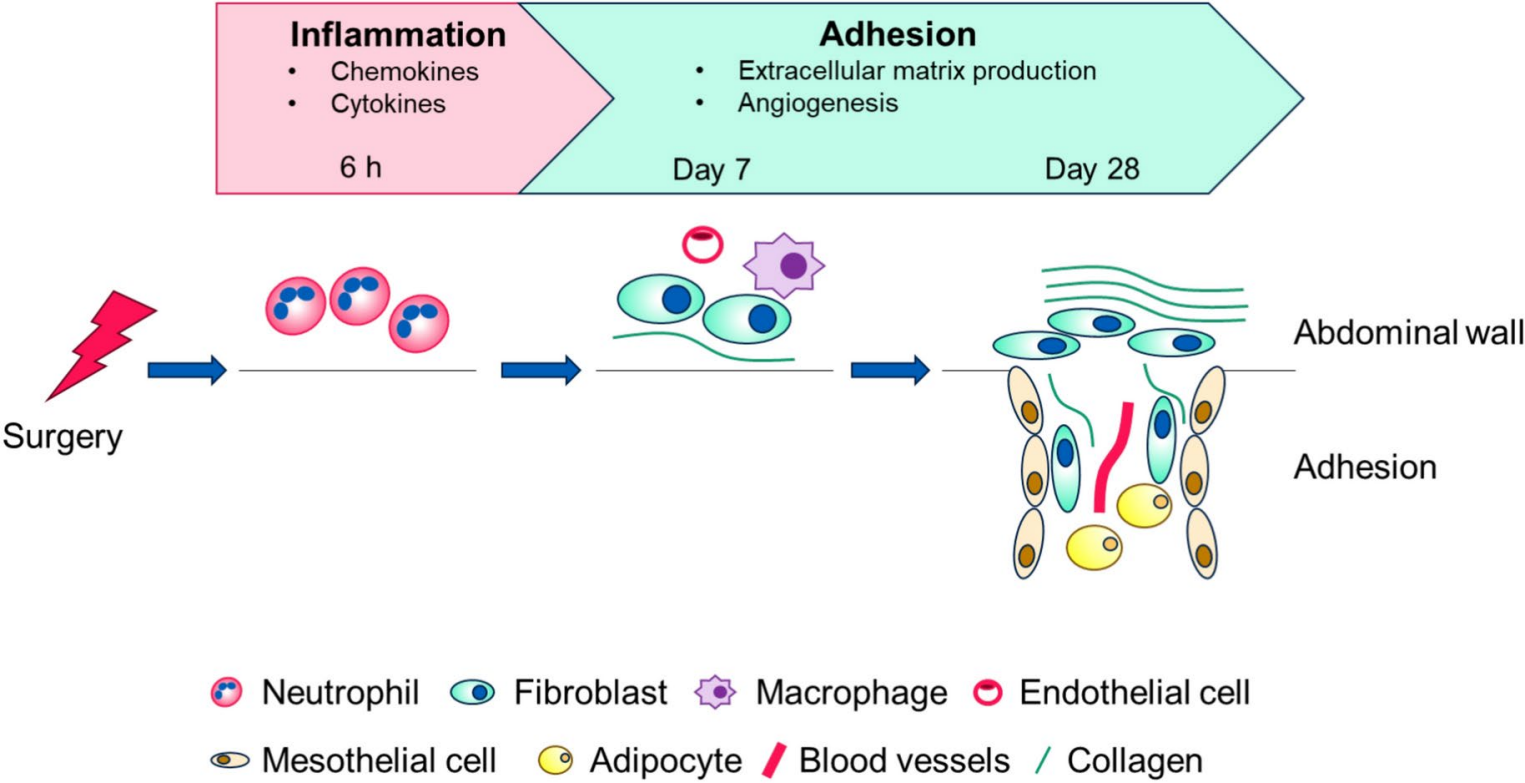
Time course changes in the monkeys following PA induction surgery**.** 6 h after the surgery, inflammatory responses including infiltration of neutrophils and production of cytokines and chemokines were upregulated. At Day 7, fibroblasts producing collagen, macrophages and endothelial cells were present at the injured abdominal walls. At Day 28, collagen producing fibroblasts, blood vessels, and adipocytes were present in the adhesion area.

Given that PA typically originate from sites of tissue injury, we evaluated the validity of the PA induction surgery by comparing the injury sites of monkeys following PA induction surgery with those following C-sections. Both groups exhibited similar time-course changes, with neutrophil infiltration occurring at the early time point (6 h), followed by the presence of collagen-producing fibroblasts at the injury sites (day 7 and day 28 or 30). These results suggest that this PA induction surgery mirrors the response observed after open abdominal surgery. At day 7, we confirmed that CD31-positive endothelial cells and CD68-positive macrophages were present at the surgery sites. These data were consistent with the reported PA progression cascade, wherein neutrophils infiltrate the wound area, followed by an increase in macrophages, fibroblasts, and blood vessels^7,8^. Histopathological evaluation of the adhesion area confirmed that it consisted of collagen-producing spindle-shaped cells (considered to be fibroblasts), blood vessels, and adipocytes. Histopathological analyses of human peritoneal adhesions have been reported similar components, blood vessels, adipose tissues, and smooth collagen-lined muscle cell clusters^20^. Furthermore, recent research has highlighted the importance of mesothelin-expressing mesothelial cells in PA progression, present in both experimental mouse PA models and human adhesion areas^21^. Notably, we detected mesothelin-positive cells in the adhesion areas in the monkeys following PA induction surgery. These results indicate that our model not only induces histopathological changes similar to those observed in open abdominal surgery in monkeys, but also closely reflects the pathophysiology of human PA, demonstrating high translatability to human biology. Figure 5 illustrates the time-course changes in the monkeys following PA induction surgery**.**

We also conducted RNA-seq analysis to evaluate time-course transcriptional changes. PA progression is thought to follow a cascade: (1) surgical trauma to tissue induces the production of inflammatory cytokines or chemokines and the infiltration of inflammatory cells like neutrophils and macrophages, (2) fibrin deposition occurs due to an imbalance between fibrin formation and fibrinolysis, (3) ingrowth of fibroblasts and blood vessels^6,7^. Enrichment analyses using GO and Hallmark gene sets revealed the upregulation of inflammation-related genes at 6 h, followed by extracellular matrix remodeling genes at day 7. Additionally, we confirmed the upregulation of the *SERPINE1* (PAI-1) gene at 6 h, which plays a central role in fibrin formation. This is consistent with its upregulation in experimental PA models^9,22^. These results suggest that our PA induction surgery successfully triggered the known PA progression cascade.

Enrichment analyses using Hallmark gene sets revealed that TNF-α, IL-6, and IFN-γ signaling pathways were highly upregulated at 6 h after surgery, all of which are reported to be involved in PA progression^9,10,23^. Interestingly, IL-8, a well-known neutrophil chemokine that is absent in rodents^19^, was found to be significantly upregulated at 6 h after surgery. Although some review articles have indicated the involvement of IL-8 in PA as a chemoattractant for neutrophils^24,25^, the exact role of IL-8 in adhesion formation has never been fully elucidated. CXCL1 and CXCL2, functional homologues of IL-8 in rodents^11^, have been reported to induce the migration of neutrophils to the surgical area, thereby promoting the formation of PA^9,26^. However, the roles of CXCL1 and CXCL2 may differ in humans, as IL-8 is considered the most influential chemokine in human inflammatory responses^19^. Indeed, in a study using monkeys with surgically induced endometriosis, we previously observed that anti-IL-8 antibody not only improved the endometriosis itself but also reduced PA caused by its induction surgery, indicating that IL-8 is a potential novel target for PA treatment^27^. Our novel PA model enables the evaluation of targets that could not be assessed in rodent models due to genetic differences between humans and rodents, such as in IL-8. In the future, further evaluation of IL-8 using this model may contribute to the development of novel therapeutic strategies for PA prevention and treatment.

Genomic differences between rodents and humans are one of the factors that widen the gap between preclinical and clinical research. Cynomolgus monkeys share close similarities with humans both in anatomy and genomics, making them likely to yield highly translatable insights into human biology. We demonstrated that our monkey PA model not only reliably and reproducibly establishes PA but also mirrors human PA characteristics. This indicates its usefulness as an animal model for PA research and suggests high translatability to human biology. In conclusion, our novel surgical model, which has the potential to overcome the limitations of rodent models in terms of human translatability, provides a promising tool for elucidating PA mechanisms and assessing new therapeutic treatments with a higher probability of clinical success.

## Supplementary Information

Supplementary Information.

## Data Availability

The RNA-seq data are available in the DNA Data Bank of Japan (DDBJ) repository with accession number PRJDB19007. Other datasets generated during and/or analyzed during the current study are available from the corresponding author on reasonable request.
