# Supplementary material for: Development of a novel postoperative adhesion induction model in cynomolgus monkeys with high reliability and reproducibility
Source: Sci Rep. 2025 Feb 27;15:7102. doi: 10.1038/s41598-025-88022-3 (PMC11868379; doi:10.1038/s41598-025-88022-3)
Supplement: Supplementary file 1 — Supplementary Information. [file 41598_2025_88022_MOESM1_ESM.pdf]

Supplementary information

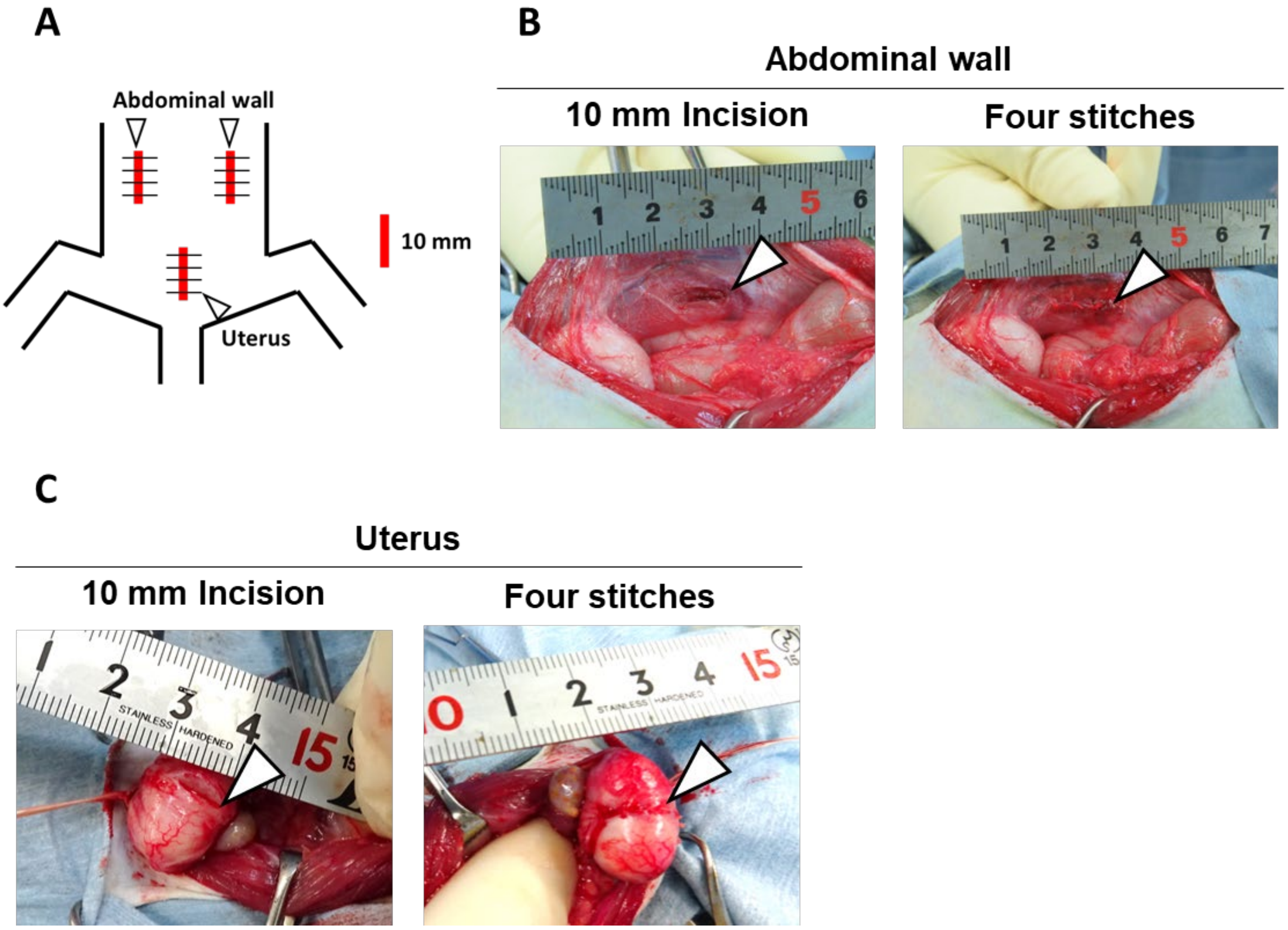

Supplementary Figure S1

Methods for surgically inducing postoperative adhesions (PA) in cynomolgus monkeys. (**A**) PA were surgically induced at the right and left abdominal walls and uterus in female monkeys. (**B**) Procedures to induce PA in abdominal walls. After a midline incision, abdominal walls about 20 mm from midline were dissected for 10 mm (Left), abraded, and sutured four times (Right). Arrowheads show the injured site. (**C**) The same procedure was also performed at the uterus in the female monkeys. Arrowheads indicate the injured site.

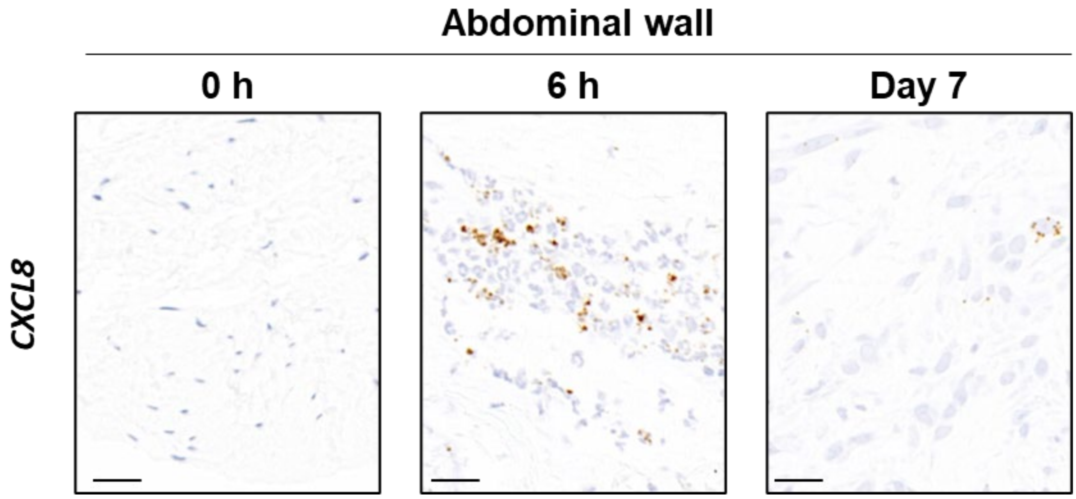

Supplementary Figure S2

Evaluation of *CXCL8* positive cells in the monkeys following PA induction surgery**.** Representative images of in situ hybridization staining for *CXCL8*. Scale bars, 20 μm.
